# Pennsylvania State Veterinary Medical Association

**Published:** 1900-01

**Authors:** 


Secretary.

### PENNSYLVANIA STATE VETERINARY MEDICAL ASSOCIATION.

THE autumn session for 1899 was held in the city of Scranton, Lackawanna County, on Tuesday, September 19th.

The local reception committee treated the members and ladies present to an early morning drive over the Scranton new boulevard and around Lake Scranton *via* Nay Aug Park. At a picturesque bend of the road on the shore of the lake Mrs. Dr. Helmer photographed the excursion party, which enabled the members to avail themselves of a memento of one of the most beautiful niches in nature that Northeastern Pennsylvania affords.

Returning to the city, dinner was partaken of at Hotel Jermyn, after which the members repaired to the Board of Trade Building, the beautiful assembly room of which had been engaged by the Committee of Arrangements for the deliberations of the convention.

At 2 P.M. the President, Dr. J. Curtis Michener, of Colmar, Pa., took the chair and called the meeting to order.

In the absence of the Recording Secretary, Dr. C. J. Marshall, Dr. Helmer, ex-Secretary, was asked to perform the duties of this office. Roll-call was responded to by the following members: Drs. J. W. Adams, J. F. Butterfield, John R. Courtright, George B. DuBois, J. C. Foelker, Jacob Helmer, W. Horace Hoskins, J. D. Houldsworth, Edward Hogg, J. Curtis Michener, Otto G. Noack, Leonard Pearson, Thomas B. Rayner, James B. Rayner, R. G. Rice, W. L. Rhoads, D. C. Stanton, James H. Timberman. Visitors were: Drs. H. R. Church, Luzerne Borough, Pa.; W. J. Fretz, Perkasie, Pa.; Charles T. Gelbert, Jr., Scranton, Pa.; L. E. Mead, Tunkhannock, Pa.; W. J. Niles, Pleasant Mount, Pa.; E. E. Tower, Montrose, Pa.; H. A. Paget, Scranton, Pa.; A. R. Potteigor, Selin's Grove, Pa.; W. J. Storm, Dunmore, Pa. A motion made at this juncture by Dr. Pearson to the effect that the reading of the minutes of the previous meeting be dispensed with, owing to the lateness of the hour and the length of the report, received a ready second and was carried.

Dr. Michener, the President, then addressed the meeting. His remarks were extempore, and were listened to with pleasure and interest.

The Corresponding Secretary read a list of names of those desiring to become members, and the Board of Censors acted upon the same, with the result of the recommendation of Dr. E. E. Tower, Montrose, Pa.; Dr. A. R. Potteiger, Selin's Grove, Pa.; Dr. S. Nichols, Wellsboro, Pa., who were then by acclamation duly elected as members of the Association. The application of Dr. J. P. Vasey was laid over, and the Corresponding Secretary was requested to inform him of the disposition of his application.

Letters of regret at not being able to attend the September session in Scranton were received from Drs. Francis Bridge, M. J. Chrisman, J. Buckham, C. R. Good, David Martin, C. Courtney McLean, S. Nichols,

W. Ratcliff, and James A. Waugh. Also a telegram was read from Dr. C. J. Marshall, of Philadelphia.

Drs. C. R. Good, of Lock Haven; David Waugh, Pittsburg, and C. Courtney McLean, of Meadville, Pa., were unanimously reinstated as members of the Association, a motion on reinstatement having been made by Dr. Rhoads, stating that the members named had paid their dues, and requested that their names be replaced upon the roll. A motion that the Committee on Resolutions write to Dr. Collins regarding the welfare of his son, who was very ill from appendicitis, was favorably received, but Dr. Rhoads explained that he had already written to Dr. Collins in reference to the condition of his son, expressing the solicitude of the Association in his rapid restoration to health.

There being present no delegates with reports, Dr. Rhoads suggested and the president asked those persons present who were delegates to the National Association, so recently held in New York, to favor the members with a talk upon their observations while in attendance.

The President called upon Dr. Hoskins, who in his usual happy way made interesting remarks, which were followed by Dr. Pearson upon the same subject.

As County Secretary for Lebanon County, Pa., Dr. M. J. Collins reported that there had been no new registrations in his county during the year. In one section there had been a slight outbreak of spinal meningitis. Tetanus also was frequent. Had cured about 50 per cent. of cases treated this summer.

Dr. J. Buckham, writing for Beaver County, reports that there have been no outbreaks of contagious diseases since the last meeting. That the breeding of horses is increasing, fewer horses are bred in this county, but are of a better grade than formerly, especially light drivers; also that there has been considerable trouble from non-professional opposition.

Dr. C. R. Shaub, for Lancaster County, reports that among the diseases which have occurred during the year tetanus has probably been the most prominent. Out of fifteen cases thirteen were in barns where tetanus had previously existed. About one-half of the cases presented wounds. In our treatment of these cases we employ corrosive sublimate. For four or five days we give each day a hypodermatic injection of hydrarg chlor. corro., two grains, sodium chloride, five grains, in distilled water from five to six drachms, then continue giving the same solution internally in quantities which contain six grains given three times daily. Give it in the food or drink. From this line of treatment have had five recoveries from six cases. More dogs suffering from rabies have been met than ever before. All were of the dumb variety. Contagious skin diseases among dogs are not as prevalent as in previous years. Parturient paresis is frequent. Using the iodide of potash treatment have lost but one out of ten cases during the year.

There were no reports offered from the Committees on Intelligence and Education, Legislation, and Sanitary Science and Police.

(To be continued.)

